# The relationship between leisure involvement and social inclusion among Chinese older adults: the mediating roles of flow experience and fear of missing out

**DOI:** 10.3389/fpsyg.2026.1759014

**Published:** 2026-03-24

**Authors:** Jiang Hu

**Affiliations:** Department of Physical Education, Ningbo University of Technology, Ningbo, China

**Keywords:** aging population, fear of missing out, flow experience, leisure involvement, older adults, social inclusion

## Abstract

**Introduction:**

This study examined the association between leisure involvement (LI) and social inclusion (SI) among Chinese older adults, with particular attention to the mediating roles of flow experience (FE) and fear of missing out (FoMO).

**Methods:**

Data were collected from 432 older adults aged 55 years and above through community-based surveys in Zhejiang Province, China, supplemented by online questionnaires. Validated scales were used to measure LI, FE, FoMO, and SI. Mediation analyses were conducted using the SPSS PROCESS macro with bias-corrected bootstrap procedures.

**Results:**

LI was positively associated with SI. FE showed a significant positive indirect effect, whereas FoMO showed a significant negative indirect effect.

**Discussion:**

These findings suggest that leisure involvement may promote social inclusion through differentiated emotional pathways, characterized by enhanced positive experiential states and reduced socially driven anxiety. The results highlight the potential value of community-based leisure programs in promoting emotional well-being and strengthening social participation among older adults.

## Introduction

By 2050, older adults will comprise about 14% of the global population (Gholami et al., 2024), highlighting the growing importance of promoting well-being in aging societies. Social Inclusion (SI) is associated with greater opportunities for individuals—especially vulnerable groups—to participate equally in social life and maintain a sense of belonging (Xie et al., 2020). For older adults, higher SI is linked to improved life satisfaction, social support, and mental health (Su et al., 2023). However, barriers such as the digital divide, limited health, and unequal access to resources are often associated with reduced levels of participation (Zhao and Zhu, 2024; Ramlagan et al., 2013). Enhancing SI has therefore become a key target in gerontological practice (da Costa et al., 2020).

Leisure activity is an important factor associated with well-being in later life. It is associated with stronger social networks, experiences of pleasure and accomplishment, and may contribute to SI by creating opportunities for interaction (Jung and Kim, 2017). Building on prior evidence regarding the benefits of leisure involvement (LI) (Hansdottir et al., 2022), limited research has directly examined the association between LI and SI among older adults. Existing studies rarely explore the psychological mechanisms underlying this relationship. Thus, this study aims to examine whether LI predicts SI and to clarify the underlying psychological pathways.

Despite growing recognition of leisure as a resource in later life, few empirical studies have systematically examined its underlying emotional mechanisms. In particular, the positive role of Flow Experience (FE) and the potential negative role of Fear of Missing Out (FoMO) have rarely been jointly investigated in older populations. Most FoMO research has primarily focused on adolescents and young adults (Przybylski et al., 2013; Mikami et al., 2025), creating a gap in understanding how this construct operates in later life, especially within rapidly digitalizing societies such as China.

However, emerging evidence suggests that FoMO is not exclusively a youth-related phenomenon. Research involving older adults indicates that FoMO can also be observed in later life and may be associated with emotional vulnerability and relational insecurity (Durao et al., 2023). At the same time, Socioemotional Selectivity Theory (SST) posits that as individuals perceive their future time as increasingly limited, they prioritize emotionally meaningful goals over information-seeking or social expansion (Carstensen, 2006). Empirical research further demonstrates that older adults tend to reduce information-oriented social motives and instead allocate cognitive and interpersonal resources toward maintaining positive affect and optimizing emotional regulation (Moon and Isaacowitz, 2025). Such motivational shifts may influence how FoMO manifests in older populations, potentially altering its psychological pathways and overall impact compared with younger groups.

Taken together, these perspectives suggest that leisure involvement may be linked to social inclusion through differentiated emotional routes: a positive experiential pathway characterized by flow, and a potentially anxiety-related pathway involving FoMO. To clarify these mechanisms, the present study proposes and tests a dual-path mediational model.

### Leisure involvement

Leisure Involvement (LI) refers to the level of psychological and behavioral engagement individuals invest in leisure activities (Kyle et al., 2003). LI is associated with better physical and mental health, broader social networks, and greater self-expression and life satisfaction, broader social networks, and higher levels of self-expression and life satisfaction (Yueh and Chang, 2024). Active Aging Theory emphasizes that participation in leisure supports social participation and mental well-being in older adults (Fries, 2012). Through shared activities, LI is associated with greater belongingness and lower levels of loneliness (Mümken et al., 2024). From a social identity perspective, involvement in interest-based groups fosters collective identity and social inclusion (Tajfel and Turner, 2004). Prior evidence shows that older adults gain identity and inclusion through leisure groups and community clubs (Wu et al., 2023). Thus, LI is expected to predict higher SI.

*H1*: LI positively predicts SI.

### The mediating role of flow experience

Flow Experience (FE) refers to a state of deep engagement characterized by concentration, immersion, and loss of self-awareness (Csikszentmihalyi, 1990). FE has been associated with enhanced intrinsic motivation, creativity, and subjective well-being (Tian and Ou, 2023). Leisure activities that optimally balance individual skills and situational challenges are particularly conducive to the emergence of flow states. Research using experience sampling methods demonstrates that flow occurs in the everyday lives of older adults and is closely related to leisure context and social settings (Heo et al., 2010).

For older adults, participation in meaningful leisure activities is associated with improved social interaction and lower levels of anxiety and loneliness, thereby creating favorable conditions for experiencing flow (Lee et al., 2021; Stebbins, 2015). Beyond its intrapersonal benefits, empirical evidence suggests that flow experience is positively associated with participative and sharing behaviors in social contexts (Zheng, 2023), indicating that individuals experiencing flow are more likely to engage actively with others.

Within the framework of socioemotional development in later life, older adults increasingly prioritize emotionally meaningful and satisfying interactions (Carstensen, 2006; Moon and Isaacowitz, 2025). In this context, the positive affect and sense of competence generated during flow experiences may enhance perceived self-efficacy, improve the quality of social interaction, and reinforce feelings of belonging within social groups. Through these psychological pathways, flow experiences may contribute to stronger social integration and a greater sense of social inclusion. Accordingly, FE may function as a positive emotional pathway linking LI to SI.

*H2*: FE plays a mediating role between LI and SI.

### The mediating role of fear of missing out

Fear of Missing Out (FoMO) reflects anxiety about missing rewarding social experiences and the desire to remain continually connected with others (Przybylski et al., 2013). Although initially conceptualized in the context of adolescents and young adults, FoMO has increasingly been observed among older adults in the context of expanding digital engagement and social media use (Elhai et al., 2021). Emerging empirical evidence indicates that FoMO in later life may be associated with emotional vulnerability, relational insecurity, and reduced psychological well-being (Durao et al., 2023). FoMO has also been linked to heightened negative affect, lower emotional stability, and decreased life satisfaction (Brailovskaia et al., 2023).

From the perspective of Self-Determination Theory, unmet psychological needs for autonomy, competence, and relatedness may intensify FoMO (Deci and Ryan, 2000). Leisure involvement, when intrinsically motivated, may help satisfy these needs and thereby reduce socially driven anxiety. However, when leisure participation is externally motivated or accompanied by upward social comparison, individuals may become more sensitive to others’ experiences, potentially amplifying FoMO (Argan et al., 2024; Crusius et al., 2022).

Psychologically, elevated FoMO may be associated with lower levels of social functioning through several mechanisms. Persistent concern about exclusion may increase social comparison tendencies and attentional focus on perceived deficits, thereby reducing satisfaction with one’s own participation experiences. In addition, FoMO-related anxiety may be associated with poorer emotion regulation and decreased perceived social belonging, ultimately weakening individuals’ sense of inclusion within social groups. In older adults, who increasingly prioritize emotionally meaningful interactions, such anxiety-driven engagement may be particularly misaligned with their motivational goals, potentially diminishing the positive impact of leisure involvement on social integration. Accordingly, FoMO may operate as a negative emotional pathway linking LI to SI.

*H3*: FoMO plays a mediating role between LI and SI.

**Figure 1:**
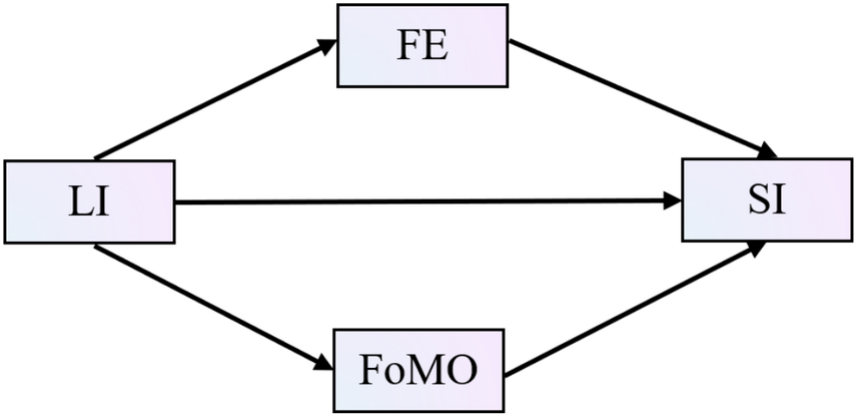
Mediation model of FE and FoMO in LI with SI. LI = Leisure Involvement, FE = Flow Experience, FoMO = Fear of Missing Out, SI = Social Inclusion.

This study proposes and tests a dual-path mediation model (Figure 1) to examine whether LI predicts SI in older adults and to test the mediating roles of FE and FoMO.

## Methods

### Research sample

To ensure adequate statistical power and enhance representativeness, this study employed a mixed-mode data collection strategy combining offline and online survey methods. The use of both modes aimed to reduce potential sampling bias associated with digital access while increasing coverage of community-dwelling older adults.

Offline questionnaires were administered in six residential communities in Zhejiang Province, China, through collaboration with local community committees. Participants were recruited at community activity centers, parks, and organized social activity venues commonly attended by older residents. Trained research assistants introduced the study purpose, obtained informed consent, and provided assistance when necessary to minimize response difficulties. A total of 258 valid offline questionnaires were collected (59.72% of the final sample).

Online questionnaires were distributed via eight community-based WeChat groups composed primarily of local residents aged 55 and above. Group administrators were contacted in advance and granted permission for survey dissemination. The online sample yielded 174 valid responses (40.28% of the final sample). The questionnaire content and structure were identical across modes to ensure measurement equivalence. Prior to data integration, responses were screened for completeness, extreme patterns, and logical consistency. Independent sample t-tests and chi-square tests were conducted to compare offline and online respondents on key demographic variables (age, gender, education level), and no statistically significant differences were observed (all *p* > 0.05), supporting the appropriateness of merging the datasets for subsequent analyses.

All questionnaires were administered with participants’ informed consent. The study was conducted in accordance with the ethical principles of the Declaration of Helsinki. Personal information was anonymized and treated with strict confidentiality.

The inclusion criteria required participants to be aged 55 years or older and residing in the selected communities. A total of 432 valid questionnaires were retained for analysis, including 203 male respondents (47.00%) and 229 female respondents (53.00%). Detailed demographic characteristics are presented in Table 1.

**Table 1:** Demographic data.

Variable	Category	Total	Percentage	Male	Male percentage	Female	Female percentage
Age group (Years)	55–64	150	34.72%	70	34.48%	80	34.93%
	65–74	160	37.04%	75	36.95%	85	37.12%
	75 and above	122	28.24%	58	28.57%	64	27.95%
Education level	Primary or below	230	53.24%	110	54.19%	120	52.40%
	Junior High	150	34.72%	68	33.50%	82	35.81%
	High School or above	52	12.04%	25	12.31%	27	11.79%
Marital status	Married	310	71.76%	145	71.43%	165	72.05%
	Widowed	100	23.15%	48	23.65%	52	22.71%
	Single/Divorced	22	5.09%	10	4.93%	12	5.24%

Based on standard sample size estimation for large populations (Fink, 2013), a minimum sample of 384 participants is required at a 95% confidence level with a 5% margin of error. The obtained sample of 432 respondents therefore satisfied this criterion.

The lower age threshold of 55 was determined based on China’s statutory retirement policy and the associated lifestyle transition from employment-centered routines to increased leisure and social engagement (Zhang et al., 2023a). Although international gerontological standards often define older adulthood as beginning at age 60 or 65, the inclusion of individuals aged 55–59 reflects the socio-institutional context of retirement in China and captures a transitional cohort experiencing shifts in social roles and digital engagement.

Given potential heterogeneity within this age range, a sensitivity analysis was conducted comparing participants aged 55–64 and those aged 65 and above (see Table 2). The results indicated no statistically significant differences across the key study variables (all *p* > 0.05), suggesting that inclusion of the younger subgroup did not materially influence the overall findings. In addition, age was statistically controlled in subsequent analyses to reduce potential cohort-related variance.

**Table 2:** Sensitivity Analysis Comparing Participants Aged 55–64 and 65 Years and Above.

**Variable**	**Age 55–64 (*n* = 150), M ± SD**	**Age ≥65 (*n* = 282), M ± SD**	***t***	***p***
LI	44.60 ± 5.30	43.80 ± 5.35	1.72	0.086
FE	43.70 ± 6.60	42.90 ± 6.75	1.55	0.122
FoMO	27.90 ± 6.40	26.80 ± 6.45	1.88	0.061
SI	59.90 ± 6.00	58.90 ± 6.20	1.64	0.101

*N* = 432, LI = Leisure involvement, FE = Flow experience, FoMO = Fear of missing out, SI=Social Inclusion.

### Activity involvement scale

The *Activity Involvement Scale* was developed by Kyle et al. (2003). Xie et al. (2022) translated and revised it as the Chinese version. This study adopted the revised Chinese version of the *Activity Involvement Scale* by Xie et al. (2022) to measure Leisure Involvement. The scale had three subscales: Attraction (five items), Centrality (three items) and Self-expression (five items). Examples of the items are as follows: (a) ‘Leisure activity is very important to me.’, (b) ‘Leisure activity plays a central role in my life’, and (c) ‘When I participate in leisure activity, others look at me as the way I want’. Participants were asked to rate the degree to which they agreed with each item on a 5-point scale from 1 (*Strongly Disagree*) to 5 (*Strongly Agree*). The scale had 13 items, and total scores ranged from 13 to 65. A higher score means a higher level of Leisure Involvement. In previous studies, this scale has demonstrated good reliability and validity among Chinese older adults (Ma, 2022). In the present study, model fit was acceptable (*χ^2^/df* = 4.76, CFI = 0.95, NFI = 0.94, GFI = 0.91, IFI = 0.92, NNFI = 0.90, RMSEA = 0.06), and internal consistency was adequate (Cronbach’s *α* = 0.82). All standardized factor loadings were significant (*λ* = 0.70–0.81, *p* < 0.001). Composite reliability (CR = 0.842) and average variance extracted (AVE = 0.641) met recommended thresholds (see Table 3), supporting convergent validity.

**Table 3:** Confirmatory factor analysis results and convergent validity.

**Construct**	**Items**	**Standardized Loadings (λ)**	**α**	**CR**	**AVE**
Leisure involvement (LI)	13	0.70–0.81	0.82	0.842	0.641
Flow experience (FE)	12	0.78–0.90	0.92	0.925	0.804
Fear of missing out (FoMO)	10	0.68–0.79	0.88	0.886	0.518
Social inclusion (SI)	17	0.69–0.82	0.83	0.854	0.662

λ = standardized factor loading; α = Cronbach’s alpha; CR = composite reliability; AVE = average variance extracted. All standardized factor loadings were significant at *p* < 0.001. CR values exceeded 0.70 and AVE values exceeded 0.50, indicating satisfactory convergent validity.

### Flow experience scale

The *Flow Experience Scale* was developed by Voelkl and Ellis (1998). Liu and Xue (2021) translated and revised it as the Chinese version. This study adopted the revised Chinese version of the *Flow Experience Scale* by Liu and Xue (2021) to measure Flow Experience. The scale had three subscales: Control (four items), Focused attention (four items), and Intrinsic interest (four items). Examples of the items are as follows: (a) ‘My participation in leisure activities goes well.’, (b) ‘I feel like time flies when I’m involved in leisure activities.’, and (c) ‘Leisure activities appeal to me.’. Participants were asked to rate the degree to which they agreed with each item on a 5-point scale from 1 (*Strongly Disagree*) to 5 (*Strongly Agree*). The scale had 12 items, and total scores ranged from 12 to 60. High scores represented high levels of Flow. In previous studies, this scale has demonstrated good reliability and validity among Chinese older adults (Liu and Xue, 2021). In the present study, model fit was acceptable (*χ^2^/df* = 3.52, CFI = 0.91, NFI = 0.94, GFI = 0.94, IFI = 0.93, NNFI = 0.90, RMSEA = 0.05), and internal consistency was strong (Cronbach’s *α* = 0.92). All standardized factor loadings were significant (*λ* = 0.78–0.90, *p* < 0.001). Composite reliability (CR = 0.925) and average variance extracted (AVE = 0.804) exceeded recommended thresholds (see Table 3), supporting convergent validity.

### Fear of missing out scale

The *Fear of Missing Out Scale* (FoMO) was developed by Przybylski et al. (2013) and translated and revised into Chinese by Cui et al. (2024). This study adopted the revised Chinese version of the FoMO by Cui et al. (2024) to measure Fear of Missing Out. The scale is a five-point Likert scale ranging from 1 (Not at all true of me) to 5 (Extremely true of me) consisting of 10 items (e.g., “I’m afraid others will have more rewarding experiences than me.”). The total score range for the scale is from 10 to 50. The higher the score is, the stronger the FoMO will be. Cui et al. (2024) demonstrated that this scale can effectively assess Fear of Missing Out among Chinese older adults. In the current study, model fit indices were acceptable (*χ^2^/df* = 3.52, CFI = 0.90, NFI = 0.90, GFI = 0.91, IFI = 0.93, NNFI = 0.92, RMSEA = 0.08), and internal consistency was adequate (Cronbach’s α = 0.88). All standardized factor loadings were significant (λ = 0.68–0.79, *p* < 0.001). Composite reliability (CR = 0.886) and average variance extracted (AVE = 0.518) exceeded the recommended threshold of 0.50 (see Table 3), indicating acceptable convergent validity.

### Social inclusion scale

The *Social Inclusion Scale* was developed by Secker et al. (2009) and translated and revised into Chinese by Chang et al. (2023). This study adopted the revised Chinese version of the *Social Inclusion Scale* to measure Social Inclusion. The scale had three subscales: Social isolation (five items), Social relations (eight items) and Social acceptance (four items). Examples of the items are as follows: (a) ‘I have felt terribly alone and isolated.’, (b) ‘I have felt what I do is valued by others.’, and (c) ‘I have felt free to express my beliefs.’. Participants were asked to rate the degree to which they agreed with each item on a 5-point scale from 1 (*Strongly Disagree*) to 5 (*Strongly Agree*). The scale had 17 items, and total scores ranged from 17 to 85. The higher the score, the more socially inclusive. In previous studies, Ilgaz et al. (2019) demonstrated that this scale is effective for research involving older adult populations. In this study, model fit was acceptable (*χ^2^/df* = 3.81, CFI = 0.90, NFI = 0.89, GFI = 0.90, IFI = 0.94, NNFI = 0.91, RMSEA = 0.07), and internal consistency was satisfactory (Cronbach’s *α* = 0.83). All standardized factor loadings were significant (*λ* = 0.69–0.82, *p* < 0.001). Composite reliability (CR = 0.854) and average variance extracted (AVE = 0.662) met recommended criteria (see Table 3), supporting convergent validity.

### Discriminant validity

Discriminant validity was examined using the Fornell–Larcker criterion. The square roots of AVE for each construct were greater than all corresponding inter-construct correlations (see Table 4), indicating adequate discriminant validity among the four constructs.

**Table 4:** Discriminant validity.

**Variable**	**LI**	**FE**	**FoMO**	**SI**
LI	**0.801**			
FE	0.64	**0.897**		
FoMO	−0.15	−0.04	**0.72**	
SI	0.43	0.36	−0.21	**0.814**

Diagonal values represent the square roots of AVE. The bold values represent the square roots of AVE.

### Data analysis

This study employed a cross-sectional research design. All statistical analyses were conducted using SPSS (Version 27.0) and AMOS (Version 28.0). Descriptive statistics and Pearson’s correlation analyses were performed to examine associations among the study variables.

Confirmatory Factor Analysis (CFA) was conducted to assess the measurement model. Convergent validity was evaluated using standardized factor loadings (λ), Composite Reliability (CR), and Average Variance Extracted (AVE). Factor loadings above 0.50, CR values above 0.70, and AVE values above 0.50 were considered acceptable. Discriminant validity was examined using the Fornell–Larcker criterion, whereby the square root of AVE for each construct was compared with inter-construct correlations. To examine potential multicollinearity, Variance Inflation Factor (VIF) values were calculated for all regression models. VIF values below 5.0 were considered indicative of acceptable collinearity levels.

To test the proposed mediation model, PROCESS Macro (Model 4; Hayes, 2013) was applied. Leisure Involvement (LI) was specified as the independent variable, Social Inclusion (SI) as the dependent variable, and Flow Experience (FE) and Fear of Missing Out (FoMO) as parallel mediators. Gender and age were included as control variables. Indirect effects were estimated using bias-corrected bootstrap procedures with 5,000 resamples. Mediation effects were considered statistically significant when the 95% confidence interval did not include zero.

## Results

### Common method variance test

To evaluate the distinctiveness of the study constructs, confirmatory factor analyses (CFA) were conducted to compare the hypothesized four-factor measurement model (Leisure Involvement, Flow Experience, Fear of Missing Out, and Social Inclusion) with several alternative models. Specifically, we tested (a) a one-factor model in which all items loaded onto a single latent factor, (b) a three-factor model in which conceptually related constructs were combined, and (c) the proposed four-factor model. Model fit was assessed using multiple indices, including *χ*^2^/*df*, CFI, TLI, RMSEA and SRMR. The four-factor model demonstrated superior fit compared with the alternative models, supporting the discriminant validity of the four constructs (see Table 5).

**Table 5:** Confirmatory factor analysis results for competing measurement models.

Model	Description	*χ^2^/df*	CFI	TLI	RMSEA	SRMR
M1	One-factor model	5.10	0.78	0.76	0.094	0.082
M2	Three-factor (LI + FE combined)	3.45	0.88	0.87	0.076	0.061
M3	Four-factor (hypothesized)	2.85	0.92	0.91	0.066	0.054
M4	Four-factor + CLF	2.78	0.925	0.915	0.065	0.052

To further address potential concerns regarding common method variance (CMV) in cross-sectional self-report data, a common latent factor (CLF) approach was implemented within the CFA framework. An additional latent factor representing common method variance was specified to load onto all observed indicators, with loadings constrained to equality for model identification. The fit of the CLF model was compared with that of the baseline four-factor model. The inclusion of the CLF did not substantially improve model fit (*ΔCFI* < 0.01), and the pattern and significance of structural relationships remained stable. These findings suggest that common method variance was unlikely to materially affect the validity of the results.

### Preliminary analysis

Descriptive statistics and correlations for the research variables are presented in Table 6. Descriptive statistics of gender, LI, FE, FoMO and SI and Pearson correlation analysis showed as follows. There is a significant positive correlation between LI and SI (*r* = 0.43, *p <* 0.001), a significant positive correlation between LI and FE (*r* = 0.64, *p < 0.001*), and a significant negative correlation between LI and FoMO (*r* = −0.15, *p <* 0.001). There is a significant positive correlation between SI and FE (*r* = 0.36, *p <* 0.001) and a significant negative correlation between SI and FoMO (*r* = −0.21, *p <* 0.001). There was no significant correlation between FE and FoMO (*r* = −0.04, *p >* 0.05). In addition, there was a positive correlation between gender and FoMO (*r* = 0.23, *p <* 0.001), while there was no significant correlation between LI, FE, SI and gender. The independent sample T test found that FoMO had a significant difference in gender [*t*_(430)_ = −4.82, *p <* 0.001], indicating that gender should be included in the model test as a control variable.

**Table 6:** Descriptive statistics and correlation table.

**Variable**	**M *±* SD**	**1**	**2**	**3**	**4**	**5**
1. Gender	1.47 *±* 0.50	1				
2. LI	44.07 ± 5.33	−0.02	1			
3. FE	43.08 ± 6.72	−0.09	0.64***	1		
4. FoMO	27.3 ± 6.43	0.23***	−0.15**	−0.04	1	
5. SI	59.33 ± 6.12	0.08	0.43***	0.36***	−0.21***	1

*N* = 432, *p < 0.05, **p < 0.01, ***p < 0.001, LI = Leisure involvement, FE = Flow experience, FoMO = Fear of missing out, SI=Social Inclusion.

### Mediating effect analysis

To examine the mediating roles of Flow Experience (FE) and Fear of Missing Out (FoMO), mediation analyses were conducted using PROCESS Macro Model 4 in SPSS (Hayes, 2013). Leisure Involvement (LI) was specified as the independent variable, Social Inclusion (SI) as the dependent variable, FE and FoMO as parallel mediators, and gender as a control variable. Indirect effects were estimated using bias-corrected bootstrap procedures with 5,000 resamples.

Prior to model estimation, multicollinearity diagnostics were conducted using variance inflation factor (VIF) statistics. All VIF values ranged from 1.12 to 1.89, which were well below the recommended threshold of 5.0 (and below the more conservative criterion of 3.0), indicating that multicollinearity did not threaten the stability of regression estimates.

**Figure 2:**
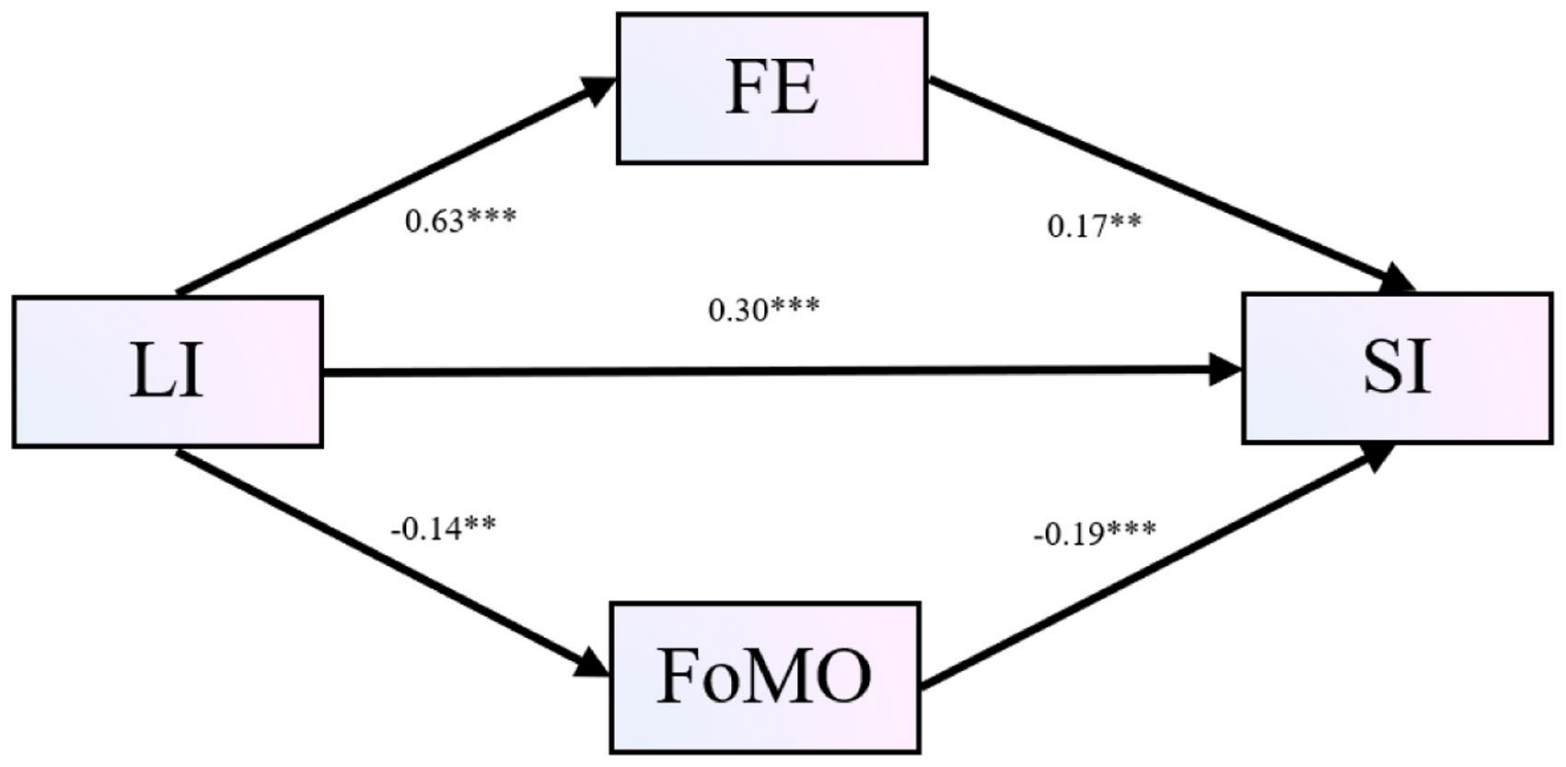
Mediating effect of FE and FoMO. *p* < 0.01, *p* < 0.001. LI = Leisure involvement, FE = Flow experience, FoMO = Fear of missing out, SI = Social inclusion.

After controlling for gender, LI was positively associated with FE (*β* = 0.63, *p* < 0.001) and negatively associated with FoMO (*β* = −0.14, *p* < 0.01). When predicting SI, LI (*β* = 0.30, *p* < 0.001) and FE (*β* = 0.17, *p* < 0.01) were positively associated with SI, whereas FoMO was negatively associated with SI (*β* = −0.19, *p* < 0.001). The total effect of LI on SI was significant (*β* = 0.44, *p* < 0.001). After including FE and FoMO in the model, the effect of LI on SI decreased (from *t* = 10.05 to *t* = 5.40), suggesting partial mediation (see Table 7). The overall model is presented in Figure 2.

**Table 7:** Analysis of the mediating role of FE and FoMO.

Variable	**Equation 1: FE**		**Equation 2: FoMO**		**Equation 3: SI**		**Equation 4: SI**	
	***B***	**t**	***B***	**t**	***B***	**t**	***B***	**t**
Gender	−0.07	−1.96*	0.22	4.80***	0.14	3.31***	0.09	2.06*
LI	0.63	17.10***	−0.14	−3.04**	0.30	5.40***	0.44	10.05***
FE					0.17	3.12**		
FoMO					−0.19	−4.32***		
*R* ^2^	0.41		0.07		0.24		0.20	
*F*	148.85***		16.457***		34.12***		52.22***	

*p < 0.05, **p < 0.01, ***p < 0.001, LI = Leisure involvement, FE = Flow experience, FoMO = Fear of missing out, SI=Social inclusion.

Bootstrap analyses (see Table 8) indicated that the direct effect of LI on SI was 0.34, with a 95% confidence interval that did not include zero, confirming partial mediation. The total effect was 0.50, and the total indirect effect was 0.16, accounting for 31.20% of the total effect. Specifically, the indirect pathway through FE (LI → FE → SI) accounted for 25% of the total effect, 95% CI [0.04, 0.21], whereas the indirect pathway through FoMO (LI → FoMO → SI) accounted for 6.20% of the total effect, 95% CI [0.01, 0.06].

**Table 8:** Mediating effect analysis.

Effect	Path	Effect size	Relative effect size	95% Confidence interval	
				Lower limit	Upper limit
Total effect		0.50		0.40	0.60
Direct effect		0.34	68.80%	0.22	0.47
Total mediating effect		0.16	31.20%	0.05	0.25
Mediating effect 1	LI-FE-SI	0.13	25.00%	0.04	0.21
Mediating effect 2	LI-FoMO-SI	0.03	6.20%	0.01	0.06

LI = Leisure Involvement, FE = Flow Experience, FoMO = Fear of Missing Out, SI=Social Inclusion.

## Discussion

### The direct association between leisure involvement and social inclusion

This study confirmed that Leisure Involvement (LI) positively predicts Social Inclusion (SI), supporting hypothesis 1 and aligning with previous findings (Dattilo, 2021; Koçak and Gürbüz, 2024). According to Self-Determination Theory, LI satisfies basic psychological needs and strengthens belonging and group identity (Deci and Ryan, 2000). Leisure settings provide older adults with low-pressure opportunities for interaction and support, reducing feelings of isolation (Choi, 2022; Kim et al., 2022). From a Social Comparison perspective, engaging with others helps older adults evaluate their roles, adjust expectations, and enhance SI (Crusius et al., 2022). Community-based activities—such as sports, arts, and volunteering—promote social skills, acceptance, and intergenerational interaction (Lowe et al., 2023; Kim et al., 2023; Standridge et al., 2020), contributing to inclusive community development.

### The mediating role of flow experience

Results show that Flow Experience (FE) significantly mediates the relationship between LI and SI (H2), consistent with earlier work suggesting leisure promotes positive emotional states and interpersonal connections (Somjee, 2024; Zhou et al., 2023). Notably, the magnitude of the indirect effect through FE was substantially larger than that of FoMO, indicating that positive experiential processes constitute the predominant psychological mechanism linking leisure involvement to social inclusion in later life.

Flow experience enhances belonging and trust, supporting social integration. Leisure activities that match skills and challenges—such as dancing, calligraphy, and tai chi—facilitate flow, improving emotional satisfaction and identity (Guo et al., 2019). When individuals experience flow, they report heightened intrinsic motivation, perceived competence, and focused engagement, which may strengthen self-efficacy in social participation. In social leisure contexts, this heightened engagement may improve the quality of interpersonal interaction, increase responsiveness to others, and reinforce mutual trust. Socially oriented activities further build belonging and contribute to SI. FE improves mental states, encourages proactive social behavior, and helps older adults form meaningful connections. Experiencing flow may reduce self-consciousness and social anxiety, allowing individuals to interact more naturally and confidently. In turn, such interactions may foster stable group identification and perceived inclusion within community networks.

For those experiencing loneliness or role loss, flow offers psychological restoration and motivation to engage (Chang, 2017). Within the framework of socioemotional development in later life, older adults increasingly prioritize emotionally meaningful and satisfying experiences. The positive affect and sense of mastery generated during flow experiences may therefore align closely with age-related motivational goals, reinforcing sustained participation and deepened social ties. Overall, FE serves as a robust positive emotional pathway through which LI is associated with enhanced SI.

### The mediating role of fear of missing out

FoMO reflects anxiety about missing others’ experiences (Zhang et al., 2023b). Examining FoMO’s mediating role helps clarify how emotional and cognitive processes shape SI. This study found that FoMO significantly mediates the LI–SI relationship (H3). However, although statistically significant, the indirect effect size was relatively small (0.03), accounting for approximately 6.2% of the total effect. This indicates that the practical contribution of FoMO to explaining social inclusion in this sample is modest. Therefore, the mediating role of FoMO should be interpreted with caution, and its statistical significance does not necessarily imply strong practical or clinical relevance.

FoMO affects social and emotional functioning in older adults (Durao et al., 2023), heightening attention to others’ social lives and increasing loneliness and exclusion (Cui et al., 2024). Because leisure provides positive emotional experiences, it may help reduce FoMO and consequently improve SI (Levasseur et al., 2016). FoMO often arises from perceived alienation and lack of connection (Yang et al., 2024). Leisure engagement reduces excessive social comparison, fosters community affiliation, and enhances SI (Wang et al., 2024). Thus, FoMO functions as a negative emotional pathway linking LI to SI, although its explanatory magnitude in this study appears limited compared with that of Flow Experience.

One possible explanation for the relatively small magnitude of the FoMO pathway can be drawn from Socioemotional Selectivity Theory (SST). SST posits that as individuals perceive their future time as limited, they increasingly prioritize emotionally meaningful goals and affect regulation over social expansion or fear of exclusion (Carstensen, 2006). In later life, individuals are more likely to focus on maintaining satisfying relationships and positive emotional states rather than seeking broad social comparison or fearing missed opportunities. Consequently, anxiety related to missing out on others’ experiences may exert a comparatively weaker influence on social inclusion among older adults than in younger populations. This age-related motivational shift may help explain why FoMO, although statistically significant, demonstrated only a modest indirect effect in the present study.

FE and FoMO represent complementary emotional mechanisms, but they differ in magnitude. FE promotes SI by nurturing positive psychological states and appears to function as the predominant pathway in this model, whereas FoMO explains how anxiety from missing social contact may undermine inclusion (Zhang and Wang, 2024). Experiencing flow helps older adults focus on meaningful engagement rather than comparison, thereby weakening FoMO. While the dual-path model illustrates that LI is associated with SI through both enhanced positive emotions and reduced negative affect, the findings suggest that positive experiential processes play a more substantial role than anxiety-related mechanisms in later life.

### Practical implications

The findings offer practical implications for policymakers and practitioners aiming to support social inclusion through leisure engagement, while recognizing that the relative strengths of the identified mechanisms differ.

First, community programs may prioritize designing leisure activities that align with older adults’ interests and functional capacities, thereby increasing the likelihood of facilitating flow experiences. Given that Flow Experience accounted for the predominant indirect effect in the present model, structured activities that balance challenge and skill—such as arts, physical exercise, or skill-based group learning—may contribute to enhanced social interaction quality and a stronger sense of belonging.

Second, although FoMO demonstrated a statistically significant mediating effect, its magnitude was comparatively modest. Therefore, interventions targeting FoMO may be considered complementary rather than central strategies. In contexts characterized by rapid digitalization, digital literacy initiatives and inclusive communication practices may help reduce anxiety related to social comparison or perceived exclusion, particularly among individuals who are more vulnerable to digitally mediated social pressures.

Third, leisure initiatives that foster psychologically meaningful engagement may be associated with improved emotional well-being and reduced loneliness. Programs emphasizing autonomy, competence, and meaningful social contact are likely to support emotional regulation goals that are especially salient in later life.

Finally, policymakers may integrate leisure involvement into broader active aging frameworks by expanding access to community-based leisure spaces, reducing participation barriers, and encouraging intergenerational interaction. When implemented thoughtfully, such strategies may be linked to improved social participation and enhanced quality of life among older adults.

### Limitations and future directions

This study has several limitations that should be acknowledged. First, although mediation analyses were conducted using PROCESS Macro (Model 4), the cross-sectional design does not allow for firm conclusions regarding temporal ordering among variables. As Maxwell and Cole (2007) caution, cross-sectional mediation models may yield biased estimates when temporal precedence is not established. Therefore, the present findings should be interpreted as reflecting statistical associations rather than definitive causal effects. It is also possible that reciprocal or reverse relationships exist—for example, higher social inclusion may be associated with greater leisure involvement. Longitudinal and cross-lagged designs would provide stronger tests of directional mechanisms.

Second, the sample was drawn from community-dwelling older adults in Zhejiang Province, which may limit generalizability to other cultural or regional contexts. Replication across diverse populations would strengthen external validity.

Third, all measures relied on self-report instruments, which may introduce common method bias and social desirability effects despite statistical checks. Future studies may incorporate multi-source data, behavioral indicators, or experience-sampling methods to enhance measurement robustness.

Finally, although Flow Experience and FoMO were examined as mediators, additional psychological and contextual variables—such as social support, self-efficacy, and resilience—may also contribute to social inclusion. Further research could extend the proposed model and examine potential moderating factors.

## Conclusion

This study contributes to a more nuanced understanding of the psychological processes linking leisure engagement to social inclusion in later life. Rather than merely demonstrating an association, the findings clarify how experiential and affective factors operate within this relationship. The results suggest that engagement in meaningful leisure contexts may facilitate adaptive emotional experiences while attenuating socially oriented concerns, thereby shaping pathways toward greater social connectedness.

These insights extend existing discussions on active aging by highlighting the importance of internal psychological mechanisms alongside structural opportunities for participation. From a practical perspective, initiatives aimed at supporting older adults should move beyond increasing activity frequency and instead consider the quality of experience and emotional context of participation. Such an approach may better foster sustainable social integration and well-being within rapidly changing social environments.

## Data Availability

The raw data supporting the conclusions of this article will be made available by the authors, without undue reservation.
